# Elevation of α-1,3 fucosylation promotes the binding ability of TNFR1 to TNF-α and contributes to osteoarthritic cartilage destruction and apoptosis

**DOI:** 10.1186/s13075-022-02776-z

**Published:** 2022-04-29

**Authors:** Hanjie Yu, Mingxiu Li, Xiaodong Wen, Jie Yang, Xiaojun Liang, Xia Li, Xiaojuan Bao, Jian Shu, Xiameng Ren, Wentian Chen, Zheng Li, Yi Li

**Affiliations:** 1 Laboratory for Functional Glycomics, College of Life Sciences, Northwest University, 229 Taibai North Road, Xi’an, 710069 Shaanxi Province China; 2 The Second Clinical Medical College of Shaanxi University of Chinese Medicine, Xianyang, China; 3 Department of Foot and Ankle Surgery, Honghui Hospital, Xi’an Jiaotong University, 76 Nanguo Road, Xi’an, 710054 Shaanxi Province China

**Keywords:** Osteoarthritis, Chondrocyte, α-1,3 Fucosylation, FUT10, TNF-α, TNFR1, Apoptosis

## Abstract

**Background:**

Osteoarthritis (OA) is the most common form of arthritis and is characterized by the degradation of articular cartilage and inflammation of the synovial membrane. Fucosylation is an important feature of protein N/O-glycosylation and is involved in a variety of pathological processes, including inflammation and cancer. However, whether fucosylation impacts the OA pathological process is unknown.

**Methods:**

Total proteins were extracted from cartilage samples obtained from patients with OA (*n* = 11) and OA rabbit models at different time points (*n* = 12). OA-associated abnormal glycopatterns were evaluated by lectin microarrays and lectin blots. The expression of fucosyltransferases involved in the synthesis of α-1,3 fucosylation was assessed by semi-qPCR. The synthesis of α-1,3 fucosylation mediated by FUT10 was interrupted by the transfection of siRNA, and the effect of α-1,3 fucosylation on OA-associated events was assessed. Then, immunoprecipitation and lectin blotting were used to investigate the relationship between the α-1,3 fucosylation level of tumor necrosis factor receptor superfamily member 1A (TNFR1) and OA. Finally, a TNFR1 antibody microarray was fabricated to evaluate the effect of α-1,3 fucosylation on the ability of TNFR1 to bind to tumor necrosis factor-α (TNF-α).

**Results:**

Elevated α-1,3 fucosylation was observed in cartilage from OA patients, rabbit models, and chondrocytes induced by TNF-α (fold change> 2, *p*< 0.01). Our results and the GEO database indicated that the overexpression of FUT10 contributed to this alteration. Silencing the expression of FUT10 impaired the ability of TNFR1 to bind to TNF-α, impeded activation of the NF-κB and P38/JNK-MAPK pathways, and eventually retarded extracellular matrix (ECM) degradation, senescence, and apoptosis in chondrocytes exposed to TNF-α.

**Conclusion:**

The elevation of α-1,3 fucosylation is not only a characteristic of OA but also impacts the OA pathological process. Our work provides a new positive feedback loop of “inflammation conditions/TNF-α/FUT10/α-1,3 fucosylation of TNFR1/NF-κB and P38/JNK-MAPK pathways/proinflammatory processes” that contributes to ECM degradation and chondrocyte apoptosis.

**Supplementary Information:**

The online version contains supplementary material available at 10.1186/s13075-022-02776-z.

## Background

Osteoarthritis (OA) is the most common form of degenerative joint disease, affecting millions of people worldwide. The hallmarks of osteoarthritic cartilage degeneration are cartilage destruction, subchondral bone remodeling, and loss of matrix molecules [1–3]. The etiology of OA is complicated, and several factors, such as age, gender, obesity, joint injuries and genetic predisposition are involved in this process; moreover, molecular alterations of chondrocytes also play pivotal roles [4–6]. During the development of OA, proinflammatory cytokines such as interleukin-1ß (IL-1β) and tumor necrosis factor-α (TNF-α) are increased in cartilage and serve as critical mediators that impair the balance between excessive cartilage damage and the cartilage repair process in arthritis [7–9]. These cytokines activate and increase the gene expression of matrix metalloproteinases (MMPs) and aggrecanases, which digest the components of the cartilage extracellular matrix (ECM), including type II collagen and aggrecan, and facilitate cartilage destruction [10, 11]. In addition, cytokines block the synthesis of the ECM by suppressing the expression of structural proteins, such as type-II collagen and aggrecan [12–14].

Glycosylation is one of the most common and complex forms of posttranslational modification. In general, glycosylation is critical for maintaining the conformational stability and functionality of proteins and is involved in many biological processes, such as the recognition of pathogens and inflammation [15–17]. Several studies have indicated that the aberrant glycosylation of chondrocytes is associated with OA. The sialylation and fucosylation of N-glycans were altered in the cartilage of OA animal models, and these alterations preceded histological changes in cartilage [18]. The level of O-GlcNAcylation is increased in OA cartilage, and the proinflammatory milieu induced by IL-1α can promote the accumulation of O-GlcNAcylated proteins in OA cartilage [19]. Despite the importance described for glycosylation in OA, the role of altered glycosylation in the pathophysiology of OA and cartilage degradation is still not fully understood.

Tumor necrosis factor receptor superfamily member 1A (TNFR1) is a member of the TNF receptor family that serves as a receptor of the TNF-α trimer and activates the canonical TNF-α signaling pathway. The death domain of TNFR1 interacts with TNF receptor-associated protein with death domain (TRADD) and recruits Fas-associated protein with death domain (FADD) and caspase-8, consequently leading to the activation of caspase-3 and triggering apoptosis [20]. In addition, TNFR1 also mediates the NF-κB signaling pathway and contributes to inflammatory activity in OA cartilage [21]. Glycosylation is vital for the function of TNFR1; N-glycosylation is critical for the binding capacity of TNFR1 to TNF-α and promotes the formation of a TNF-α autocrine loop and inflammation in microglia via NF-κB pathways [22]. However, whether the glycosylation of TNFR1 is altered in OA chondrocytes and how the altered glycosylation affects the OA process remain to be illuminated.

In the present study, knee articular cartilage from patients with OA and from OA animal models was obtained, and the altered glycosylation associated with OA was evaluated by integrated glycomics approaches. Our study demonstrates that the synthesis of α-1,3 fucosylation mediated by FUT10 was elevated in OA cartilage and chondrocytes. Silencing the expression of FUT10 evidently reduced the expression of MMP13 and IL-1β and inhibited chondrocyte apoptosis and senescence induced by TNF-α. Interestingly, our results demonstrated a high level of α-1,3 fucosylation of TNFR1 in OA chondrocytes. Silencing FUT10 expression not only depressed the α-1,3 fucosylation level of TNFR1 but also impaired the binding ability of TNFR1 to TNF-α, which is important for the activation of the TNFR1 and NF-κB pathways. Our findings may provide new clues for understanding glycosylation in the pathogenesis of OA and a new target for therapy.

## Materials and methods

### Collection of tissue specimens

Osteochondral specimens of the knee joints were obtained from operations performed from 2020 to 2021 in the Department of Bone Microsurgery, Foot and Ankle Surgery, and Joint Surgery of Xi’an Honghui Hospital. All tissue samples had a final diagnosis provided by clinical specialists. The inclusion criteria were as follows: primary knee joint OA and Kellgren-Lawrence grading of knee X-ray of iii or iv. Patients with rheumatoid arthritis, infectious arthritis, traumatic arthritis, and other immune system diseases were excluded. As a result, 12 patients were enrolled in the knee OA group, including 5 males and 7 females with an average age of 66.1 years (range, 57–75 years). All patients underwent knee replacement surgery. Meanwhile, 11 tibia platform and distal femur comminuted fracture patients were recruited into the control group (7 males and 4 females, average age of 41.3 years [range, 24-56 years]). As the fixed weight-bearing area free bone cartilage block could not be reset in all cases, the pain symptoms of the wounded knee before seeking medical help were compared with the contralateral knee by X-ray and osteoarthritic performance.

### Histologic analysis

To evaluate the degree of degradative changes in the cartilage, cartilaginous tissue from patients with OA and from OA rabbit models was embedded in paraffin and sectioned at 6 μm. After deparaffinization and hydration, the sections were stained with hematoxylin-eosin (H&E) and safranin-O (Solarbio, China) according to the manufacturer’s instructions.

### Cell culture and treatment

The human chondrocyte cell line C28/I2 (C28) was cultured in DMEM/F12 supplemented with 10% FCS (Gibco Thermo Fisher, USA), 100 U/ml penicillin (Solarbio), and 100 μg/ml streptomycin (Solarbio) in a humidified incubator at 37 °C in the presence of 5% CO_2_. Before treatment, chondrocytes were serum-starved for 12 h and then stimulated with recombinant human TNF-α (active trimer, Acrobiosystems, China) at a concentration of 40 ng/ml for 48 h, and unstimulated chondrocytes were used as controls synchronously [23]. To investigate the effect of fucosylation on the OA process, 2F-peracetyl-fucose (2FPF, EMD Millipore, Germany) was used to depress the biosynthesis of fucosylation in chondrocytes [24]. Briefly, after serum starvation for 12 h, 2FPF was added to a complete medium at a concentration of 100 μM and incubated for 72 h. After that, chondrocytes were collected and subjected to further analysis.

### Construction of rabbit OA model

Hulth's modeling method was used to establish a rabbit model of knee OA [25]. Briefly, twenty-four male New Zealand white rabbits (purchased from the Laboratory Animal Center of Xi’an Jiaotong University, China) were randomly divided into an OA group (*n*=12) and a sham-operation group (*n*=12). At the time of surgery, the rabbits were 3 to 4 months old and had body weights of 2.5 ± 0.4 kg and 2.7 ± 0.3 kg, respectively (mean ± SEM). Rabbits were anesthetized with 3% sodium pentobarbital (1 mL/kg; Sigma). Intravenous cefazolin (22 mg/kg; Harbin Pharmaceutical Group Pharmaceutical General Factory, China) was administered at the time of the surgical procedure and once every 24 h for 3 days postoperatively. A midline skin incision was made over the right knee, and a medial parapatellar incision was made through the retinaculum. The medial collateral ligament was sharply divided. A medial parapatellar arthrotomy was performed, and the patella was dislocated laterally. The vascular intraarticular fat pad was retracted and protected carefully. As the knee was flexed, the anterior cruciate ligament and the posterior cruciate ligament were transected. The knee joint was then dislocated to excise the medial meniscus. The joint was irrigated with sterile saline solution. The capsule and the synovium were then closed together with a 4.0 interrupted Vicryl, and the skin was closed. A sham procedure was performed on the right hind limb to serve as a control. For sham surgical controls, the right knees were opened as described. After dislocating the patella laterally, the knee was irrigated, but the ligaments and menisci were left intact. Postoperatively, the animals were permitted cage activity without immobilization. The animals were closely monitored for health and welfare. At weeks 0, 4, 8, and 12, rabbits from the OA (*n*=3) and sham-operated groups (*n*=3) were sacrificed by an intravenous injection of an overdose of pentobarbital to obtain cartilage samples.

### Extraction of cell/tissue proteins

The proteins from chondrocytes and cartilage tissue were extracted using RIPA Lysis Buffer (Millipore, Billerica, MA, USA) and T-PER Tissue Protein Extraction Reagent (Thermo Fisher Scientific Inc., Rockford, IL, USA) according to the manufacturers’ instructions, and 1% (*v/v*) protease inhibitor cocktail (Sigma-Aldrich) was added. The protein concentration was determined using a BCA assay (Beyotime Biotechnology, Nantong, China).

### Lectin microarray and data analysis

The manufacture of the lectin microarray and data acquisition were performed as described previously [26–28]. The proteins isolated from cells or tissue were labeled with Cy3 fluorescent dye (GE Healthcare, Biosciences, Piscataway, NJ, USA) and purified using a Sephadex-G25 column (GE Healthcare). Subsequently, 4 μg of labeled protein was applied to the lectin microarrays and incubated in the chamber at 37 °C for 3 h. After washing and centrifugation, the slides were scanned using a confocal scanner (4000B, AXON Instruments, USA). The fluorescence intensities were extracted by GenePix 7.0 software (Axon). After filtration and normalization, the parallel datasets were compared with each other based on fold changes according to the following criteria: fold changes ≥ 1.50 or ≤ 0.67 and *p* < 0.05 indicated upregulation or downregulation, respectively. Significant differences in lectin between samples were evaluated using Student’s *t test*.

### Lectin blotting

Lectin blotting was performed as described previously [28–30]. Forty micrograms of protein from OA and control samples were separated by 10% SDS–PAGE and transferred to PVDF membranes (0.22 μm Millipore, Bedford, MA, USA). After blocking, the membranes were incubated with Cy5-labeled lectins overnight at 4 °C and imaged by a STROM FluorImager (Molecular Dynamics, Sunnyvale, CA, USA). Each blot was repeated three times, and the gray value of the selected protein bands was measured by ImageJ software (NIH).

### Immunoblotting

Briefly, 10 μg of protein was separated by 10% SDS–PAGE, transferred to the PVDF membranes (Millipore, Bedford, MA, USA), and blocked with 5% (w/v) skim milk (Becton Dickinson, Franklin Lakes, NJ, USA) in TBST (TBS buffer with 0.05% Tween-20, pH 7.6) or 3% BSA in PBST (for phosphorylated antibodies) for 1 h at room temperature. The membranes were probed with primary antibodies overnight at 4 °C with shaking. The primary antibodies used in this study were as follows: (i) rabbit polyclonal anti-FUT10 (Proteintech, Wuhan, China), (ii) rabbit polyclonal anti-MMP-13 (Proteintech), (iii) rabbit polyclonal anti-COL2A1 (Proteintech), (iv) mouse monoclonal anti-IL-1β (Proteintech), (v) mouse monoclonal anti-NF-κB p65 (Proteintech), (vi) mouse monoclonal anti-phospho-NF-κB p65 (Ser536, CST), (vii) rabbit polyclonal anti-IKBA (Proteintech), (viii) rabbit polyclonal anti-phospho-IKBA (Ser32, CST), (ix) mouse monoclonal anti-P38 MAPK (Proteintech), (x) rabbit monoclonal anti-Phospho-p38 MAPK (Thr180/Tyr182, CST), (xi) mouse monoclonal anti-JNK (Proteintech), (xii) mouse monoclonal phospho-SAPK/JNK (Thr183/Tyr185, CST), (xiii) mouse monoclonal anti-caspase-8 (Proteintech), (xiv) rabbit polyclonal anti-caspase-3 (Proteintech), (xv) rabbit polyclonal anti-TNFR1 (Proteintech), and (xvi) mouse monoclonal anti-β tubulin as internal control (Abways Biotechnology, Shanghai, China). After washing three times with TBST, the membranes were incubated with horseradish peroxidase (HRP)-labeled secondary antibody (Immunoway, Jiangsu, China) for 2 h at room temperature with shaking. The membranes were visualized with Immobilon Western chemiluminescent HRP substrate (Millipore, Billerica, MA, USA).

### Isolation of RNA and semiqPCR

The total RNA from chondrocytes and cartilage tissue was extracted by TRI Reagent (Sigma) according to the manufacturer’s protocol. Then, 1 μg of total RNA was reverse-transcribed using PrimeScript™ RT Master Mix (TaKaRa, Japan), and qPCR was performed using a ViiA 7 Real-Time PCR System (Applied Biosystems, USA). SYBR Green-based three-step RT–qPCR was performed using TB Green® Premix Ex Taq™ II (TaKaRa, Japan). The primer sequences were retrieved from the online PrimerBank database (https://pga.mgh.harvard.edu/primerbank/index.html). Information on the primers is summarized in Table S1.

### Transfection of small interfering RNA

Small interfering RNA (siRNA) specific to FUT10 was designed with the coding sequences of humans by the online software DSIR (http://biodev.extra.cea.fr/DSIR/DSIR.html). The information on the siRNAs is shown in Table S2. 2'Ome-modified siRNAs and scramble siRNA (negative control) were obtained from GenePharma (Shanghai GenePharma, China). Transfection was performed using HiPerFect reagent (Qiagen, Chatsworth, CA, USA). The cells were harvested 24 h after transfection, and knockdown effects were evaluated by semiqPCR. For stimulation, TNF-α (at a final concentration of 40 ng/mL) was added to the medium after transfection for 12 h and cultured for another 48 h. After that, the cells were collected and subjected to follow-up analysis.

### Cell proliferation assay

Cell proliferation was determined by a cell counting kit (CCK-8, Yeasen, Shanghai, China). Briefly, C28 cells were seeded in 96-well plates at a concentration of 5000 cells per well and incubated for 12 h. After treatment, 10 μL of CCK8 reagent was added to each well and incubated at 37 °C for 1 h. The absorbance at 450 nm of each well was measured by a microplate reader (Bio-Tek Instruments Inc., Winooski, VT, USA). The cell proliferation rates were recorded every 12 h.

### SA-β-gal staining

Senescent cells were stained using a senescence β-galactosidase staining kit (Beyotime). Briefly, after treatment, the cells were fixed for 15 min using a stain fixative. After washing three times, 1 mL of staining solution was added to each well, and the plates were incubated at 37 °C overnight. The number of SA-β-gal-positive chondrocytes in five random fields of each well was calculated using bright-field microscopy.

### Apoptosis assay

An Annexin V Alexa Fluor 647/PI apoptosis detection kit (Solarbio) was used to analyze apoptosis. Briefly, the chondrocytes were digested with 0.25% trypsin (without EDTA, Solarbio). Then, 1 mL of complete medium was added to neutralize trypsin, and the cells were collected and resuspended in cold PBS. After centrifugation, the cells were resuspended in binding buffer, and 5 μL of Annexin V/Alexa Fluor 647 was added and incubated at room temperature for 5 min in the dark. Then, 10 μL of PI was added. The cells were analyzed on a flow cytometer (ACEA Biosciences, San Diego, CA, USA), and the percentages of apoptotic cells were obtained using NovoExpress software.

### Immunoprecipitation

Immunoprecipitation was performed using protein A/G PLUS-Agarose (Santa Cruz, Santa Cruz Biotechnology, Santa Cruz, CA, USA) according to the manufacturer’s protocol with modifications. To protect the binding activity of TNFR1, low pH elution buffer (100 mM glycine, 50 mM Tris-HCl, and 500 mM NaCl, pH 2.0) was used to elute TNFR1, and 10 μL of Tris buffer (1 M, pH 9.5) was added to neutralize the low pH condition.

### Manufacture of the antibody microarray

To investigate the effect of α-1,3 fucosylation on the binding capability of TNFR1 to TNF-α, a TNFR1 antibody microarray was fabricated. The antibodies were diluted with printing buffer (PBST with 0.01% (*w/v*) BSA) to a concentration of 200 ng/μL and spotted using an arrayer (SmartArrayer 48, Capitalbio). After blocking, the enriched TNFR1 from different sources was diluted to 0, 5, 10, and 20 ng/μL, applied to microarrays, and incubated at 25 °C overnight. Then, the slides were incubated with TNF-α (20 ng/μL) and 10 ng/μL of primary antibody against TNF-α (Cy3 labeled, Bioss) for 3 h sequentially. The net fluorescence intensities (the raw fluorescence intensities—background) of each spot were acquired by Genepix 7.0 software (Axon Instruments, Inc., USA). We set the binding signals at 0 ng/μL TNFR1 as the baseline, and the differences in binding signals of TNFR1 Ab between TNF-α-treated chondrocytes, siRNA-FUT10-transfected chondrocytes, and controls were tested by one-way ANOVA.

## Results

### Assessment of abnormal glycopatterns in cartilage from OA patients

**Fig. 1:**
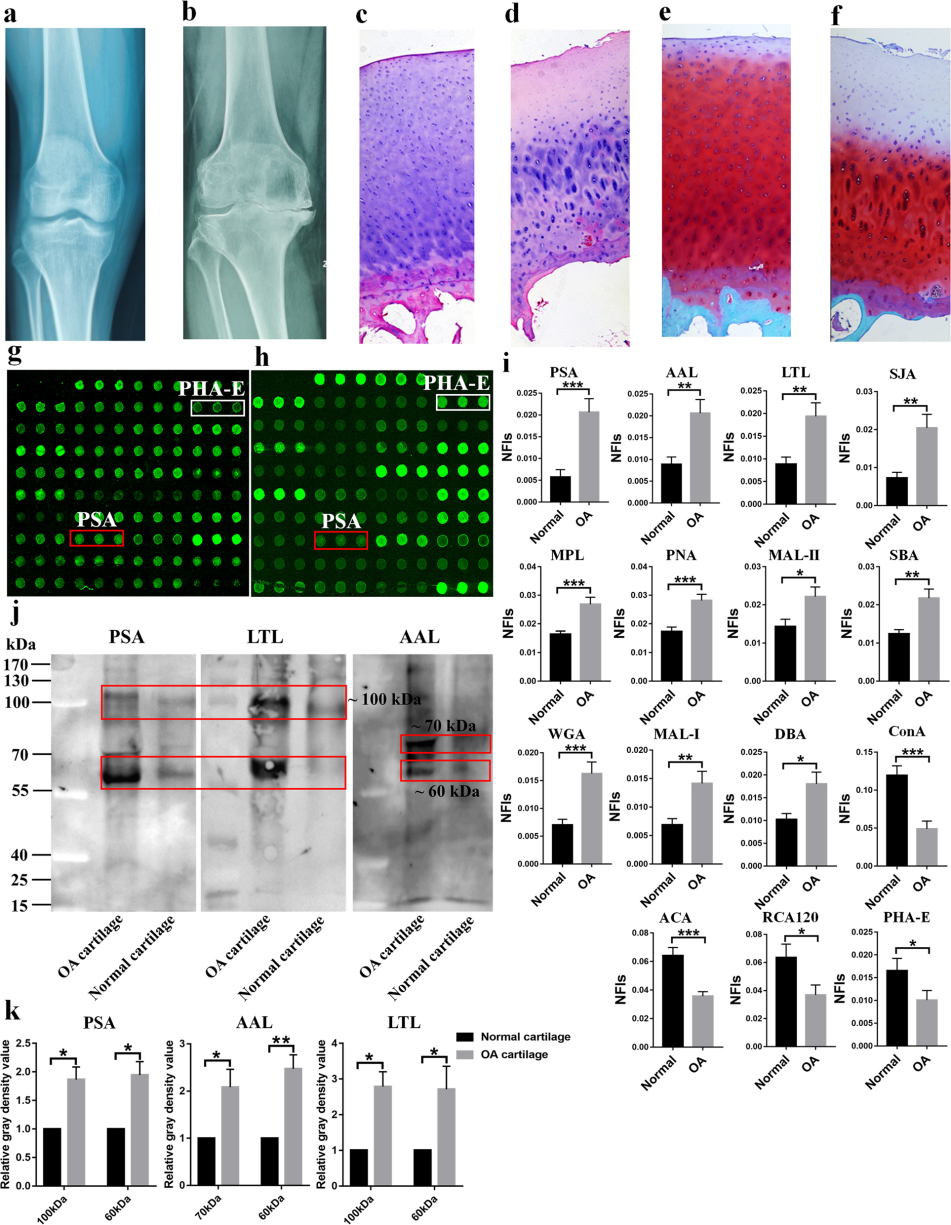
Evaluation of altered glycopatterns in cartilage from OA patients. **a**, **b** The morphology of the articular knee joint was assessed by X-ray imaging. Compared with the normal control (**a**), the joint space, and morphology of OA patients (**b**) were altered. **c**, **d** The articular cartilage from normal controls (**c**) and OA patients (**d**) was evaluated for histopathologic features using H&E staining. **e**, **f** Safranine-O staining was used to assess the degenerative degree of cartilage sections in normal control (**e**) and OA patients (**f**) (original magnification ×100). **g**, **h** Scanned images were obtained for the analysis of glycopatterns of articular cartilage from OA patients (**g**) and normal controls (**h**). **i** The lectins with increased NFIs in OA patients are marked with red boxes, and those with decreased NFIs are marked with white boxes. The NFIs of 15 lectins were significantly changed in OA patients (*n*=12) compared with normal controls (*n*=11) based on fold change and *t test*, and the data are presented as the average NFI ± SEM (*p < 0.05, **p < 0.01, and ***p < 0.001). **j** Lectin blotting of PSA, LTL, and AAL was performed to validate the differential expression of the glycopatterns in cartilage from OA patients and normal controls. The differential protein bands between OA patients and normal controls are marked with red frames. **k** The gray value of the difference protein bands was measured using ImageJ software and compared by *t test* (*p < 0.05, **p < 0.01, and ***p < 0.001)

To investigate the abnormal glycosylation associated with OA, the glycan profile of cartilage from OA patients and normal controls was evaluated by lectin microarray. As shown in Fig. 1a and b, the joint morphology and space were altered in OA patients. Moreover, abundant osteophytes, sclerosis of subchondral bone, cystic changes in the proximal medial tibia, and varus deformities were observed in the knee joints of OA patients. The staining results revealed that the OA tissue slices displayed common OA changes, including discontinuity of the cartilage surface and nonuniform distribution of chondrocytes (Fig. 1c–f). The binding of several lectins, such as *Pisum sativum agglutinin* (PSA) and *Phaseolus vulgaris agglutinin-E* (PHA-E), showed distinct differences between OA and normal cartilage, suggesting that glycosylation of cartilage was altered in OA patients (Fig. 1g, h). The average normalized fluorescent intensities (NFIs) of all lectins from OA and control samples were compared (summarized as the mean values ± SEM in Table S3). As a result, the NFIs of 15 lectins were significantly altered between OA and normal cartilage (Fig. 1i). Notably, the α-1,3/6 fucosylation levels identified by PSA, *Aleuria aurantia lectin* (AAL), and *Lotus tetragonolobus lectin* (LTL) were significantly increased in OA cartilage compared with normal controls (fold change >2, *p* <0.01).

As a result of lectin blotting, PSA and LTL showed stronger binding to two apparent bands (approximately 100 and 60 kDa), and AAL showed distinct binding to two apparent bands (approximately 60 and 70 kDa), in OA compared with control cartilage (Fig. 1j, k). These results demonstrated that glycosylation, especially α-1,3/6 fucosylation, was significantly altered in the cartilage of OA patients.

### The level of fucosylation increased in the cartilage of the OA model

**Fig. 2:**
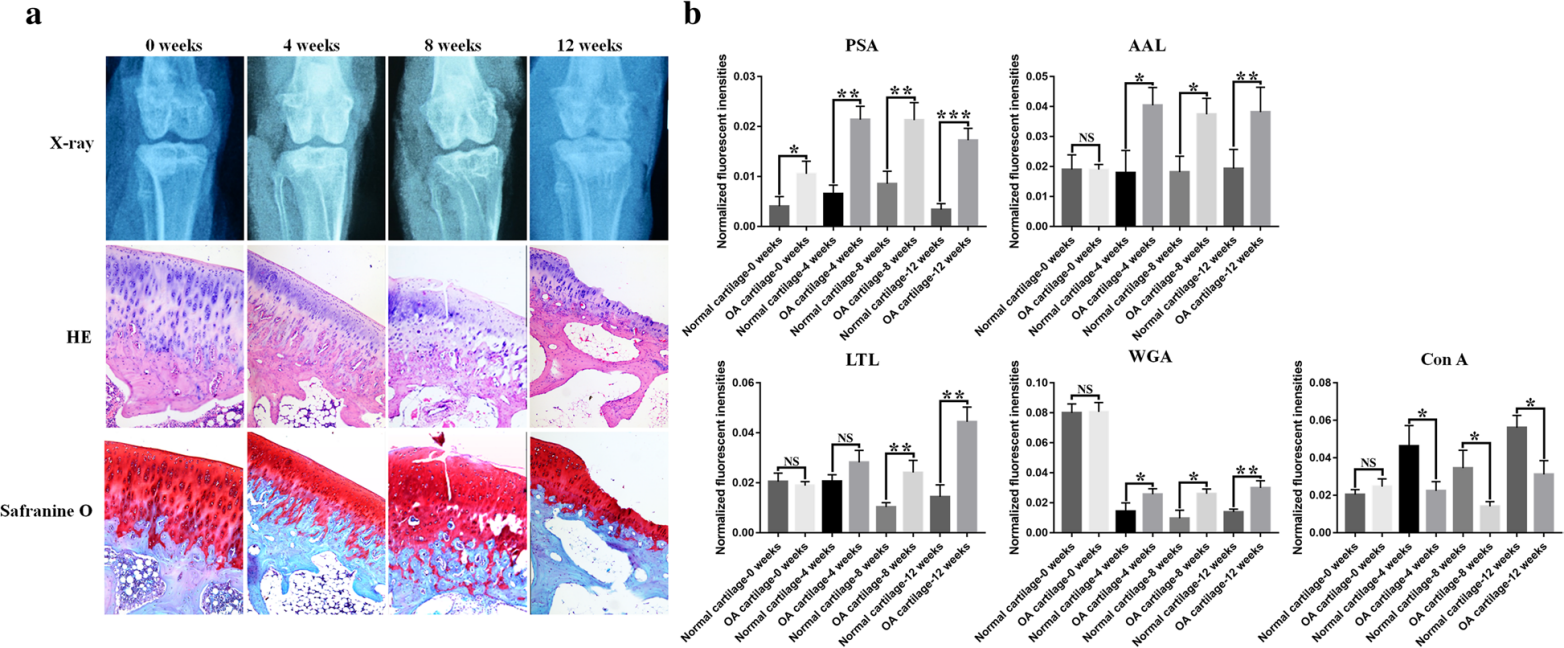
The fucosylation level was increased with the development of OA. The OA rabbit model was established to assess the dynamic change in glycopatterns in cartilage during the development of OA. **a** The X-ray image was used to evaluate the morphology of knee joints from OA models at 0, 4, 8, and 12 weeks after surgery (upper). Cartilage tissue sections were obtained from the right knee joints of OA models and stained with H&E (middle) and safranine-O (lower) for the indicated time periods (original magnification ×100). **b** The NFIs of 5 lectins were significantly altered in OA models (*n*=3 per time point) compared with sham operation controls (*n*=3 per time point) based on fold change and *t test*. (NS not significantly different, NS no significant difference, *p < 0.05, **p < 0.01, and ***p < 0.001)

During the development of OA, the medial joint space narrowed, the cartilage was slightly injured, and the cartilage surface was not smooth but rather contoured at 4 weeks. The cartilage was moderately damaged, the distribution of chondrocytes was disordered, and macrocracks on the surface of cartilage and high-density shadows were observed in joints at 8 weeks. The joint space was significantly narrowed, and osteophyte and joint deformities and advanced OA lesions, including thin hardened and rough cartilage layers, the disappearance of the abnormal tide line of subchondral bone, and severe loss of Safranine-O staining and clefts, were observed in OA rabbits at 12 weeks postoperatively (Fig. 2a).

The results of the lectin microarrays indicated that 34 lectins showed significantly altered NFIs during the OA process (Table S4). Combined with the results of OA patients (Table 1), PSA showed significantly increased NFIs in all OA models compared with the control. WGA and AAL showed significantly increased NFIs, and the high mannose-type N-glycan binder *Canavalia ensiformis Agglutinin* (ConA) showed significantly decreased NFIs in OA models 4 weeks postoperation compared with the sham-operated controls. However, the relative abundance of Fucα1-3Galβ1-4GlcNAc, which is recognized by LTL, did not show a distinct difference between OA models and controls at the early stage of OA but exhibited a significant increase in the middle and late stages of OA models compared with controls (Fig. 2b). Our findings indicated that an increased level of α1-3/6 fucosylation is associated with the OA process.

**Table 1:** The lectins showed significant altered NFIs in OA models and patients compared with their corresponding controls

Lectin	Specificity	OA models / sham-operated groups				OA patients / normal controls
		0 weeks/ control	4 weeks/ controls	8 weeks/ controls	12 weeks/ controls	
PSA	Fucα-1,6GlcNAc, α-D-Man, α-D-Glc	2.586*	3.272**	2.489**	5.104***	3.585***
AAL	α-Fucose	/	2.261*	2.064*	1.976*	2.322**
WGA	(GlcNAc)_n_ and multivalent Sia	/	1.791*	2.693*	2.155**	2.309***
LTL	Fucα1-3Galβ1-4GlcNAc, Fucα1-anti-H blood group specificity	/	/	2.339**	3.084**	2.196**
ConA	High-Mannose type N-glycans	/	0.484*	0.408*	0.556*	0.410***

/ no significant difference, * *p* < 0.05, ** *p* < 0.01, and *** *p* < 0.001

### Decreased fucosylation retarded ECM destruction

**Fig. 3:**
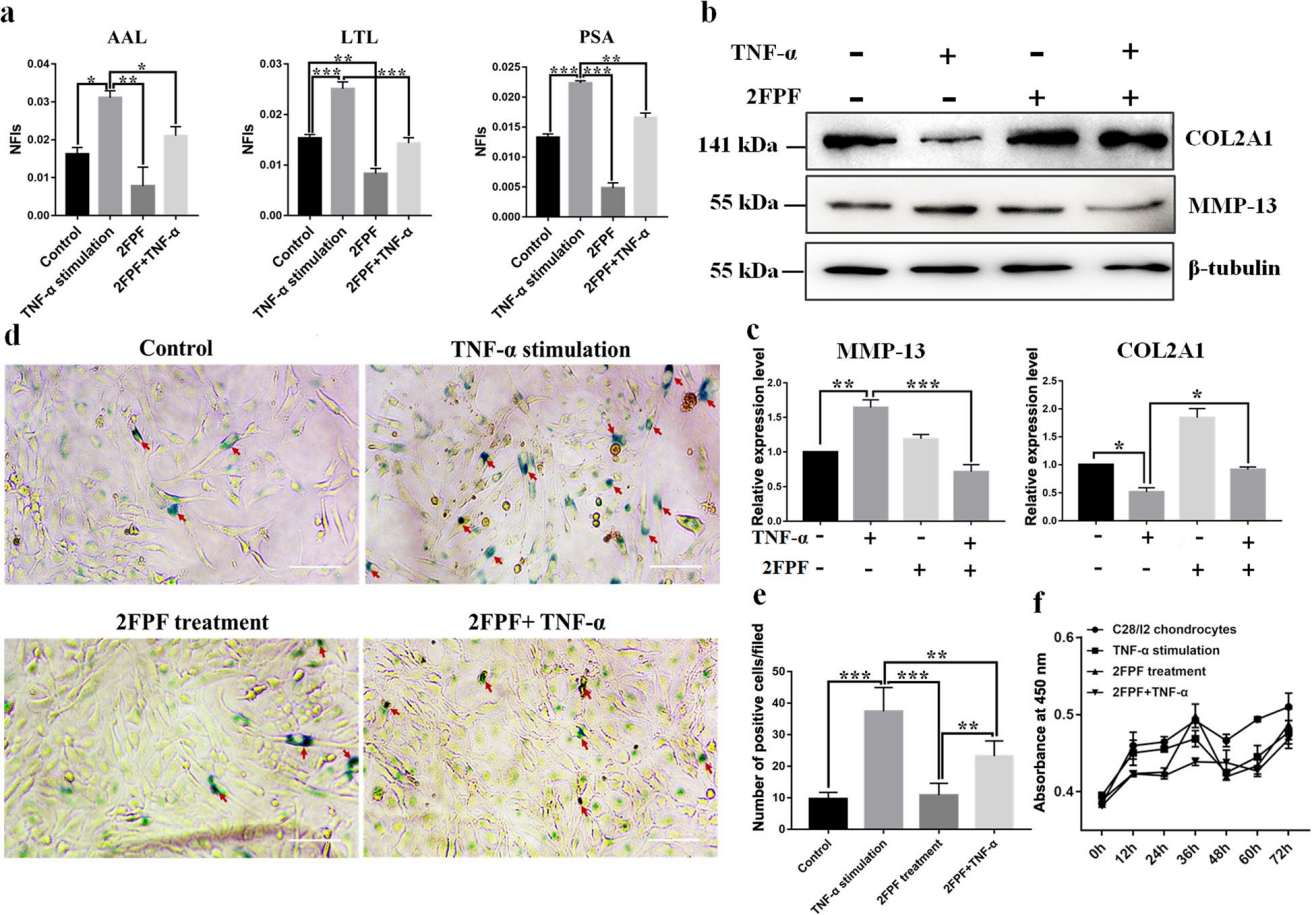
Suppression of fucosylation impeded ECM degradation and cell senescence. **a** The α-1,3/6 fucosylation levels identified by AAL, LTL, and PSA were markedly elevated in chondrocytes induced by TNF-α compared with the control and were significantly inhibited by 2FPF. **b** Effect of the fucosylation synthesis inhibitor 2FPF alone or in combination with TNF-α on the inducible expression of COL2A1 and MMP-13. β-Tubulin was used as an internal control. **c** The gray value of each protein band was extracted from three experimental replications. The expression of MMP-13 and COL2A1 was normalized to that of β-tubulin and compared between groups using one-way ANOVA. **d** SA-β-Gal staining was used to assess the effect of 2FPF on cell senescence induced by TNF-α; scale bar =20 μm. **e** The number of SA-β-Gal-positive cells in chondrocytes treated with or without TNF-α and the presence or absence of 2FPF was counted in 5 random fields. **f** The effect of 2FPF on the viability of normal and TNF-α-treated chondrocytes (*n* = 4). The data are presented as the mean ± SEM, *p < 0.05, **p < 0.01, and ***p < 0.001

Next, we primarily investigated whether fucosylation impacts OA-associated events. We used 2FPF to interrupt the synthesis of fucosylation in chondrocytes with or without TNF-α treatment. As a result, fucosylation was evidently increased in TNF-α-stimulated cells; however, the fucosylation level was suppressed significantly in chondrocytes pretreated with 2FPF (Fig. 3a). The immunoblotting results revealed that COL2A1 expression was decreased, and MMP-13 expression was increased, after treatment with TNF-α, which is a common event in OA. However, after treatment with 2FPF, the expression of COL2A1 and MMP-13 was distinctly increased or decreased compared with that in TNF-α-stimulated cells, respectively (Fig. 3b, c). Furthermore, the staining indicated that the number of SA-β-gal-positive cells was significantly increased in chondrocytes treated with TNF-α, but it was significantly decreased after treatment with 2FPF compared with TNF-α-stimulated cells (Fig. 3d, e). However, the results of the cell viability assay indicated that 2FPF did not impact chondrocyte proliferation (Fig. 3f). These findings indicated that suppression of fucosylation relieves ECM degradation and cell senescence caused by TNF-α.

### Loss of FUT10 impeded ECM degradation and apoptosis induced by TNF-α

**Fig. 4:**
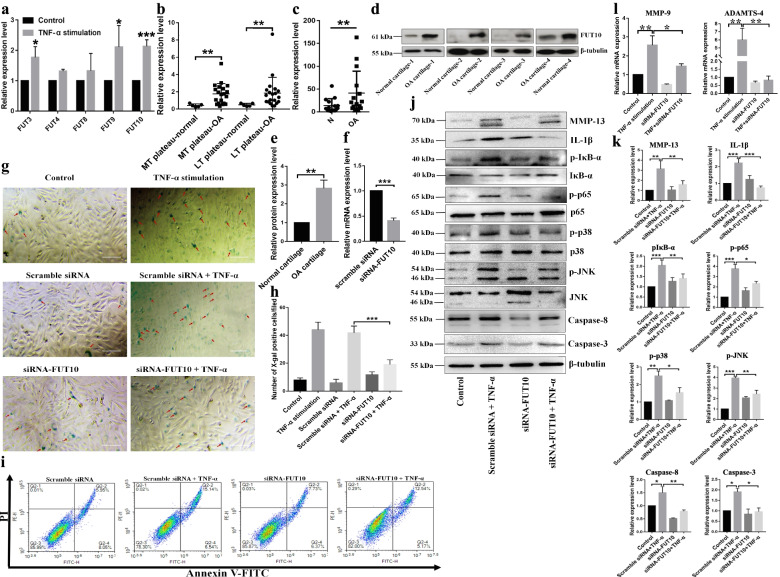
Suppression of FUT10 expression inhibited TNF-α-induced expression of OA-related proteins, senescence and apoptosis. **a** Relative expression levels of 5 FUTs involved in the synthesis of α-1,3/6 fucosylation were compared between chondrocytes treated with or without TNF-α for 48 h (*n*=3). **b** Reanalyzed gene expression data from the published database (GES51588). This finding demonstrated that the transcription level of FUT10 was significantly upregulated in the medial and lateral tibia of OA patients compared with healthy controls. **c** The relative expression level of FUT10 in OA patients and normal controls was compared using qPCR (*n*=15). **d** FUT10 expression in cartilage from OA patients and normal controls was analyzed by western blot assay. β-Tubulin was used as an internal control (*n*=4). **e** The gray value of the protein band was measured and compared using a paired *t test*. **f** The mRNA level of FUT10 was significantly decreased in chondrocytes transfected with siRNA against FUT10 compared with those transfected with scramble siRNA (*n*=4). **g** After transfection, senescent cells in chondrocytes treated with or without TNF-α were revealed by SA-β-Gal staining; scale bar =20 μm. **h** The number of SA-β-Gal-positive cells was counted in 5 random fields. The data are presented as the mean ± SEM, *p < 0.05, **p < 0.01, and ***p < 0.001. **i** After transfection, the chondrocytes were stimulated with TNF-α, and the number of apoptotic cells was determined via flow cytometry. **j** Representative immunoblot analysis of MMP-13, IL-1β, caspase-3/8, and total and phosphorylated IκB-α, p65, p38, and JNK in chondrocytes; β-tubulin was used as an internal control. **k** The relative expression levels of these proteins were obtained from three experimental replications and compared based on one-way ANOVA. **l** The relative mRNA level of MMP-9 and ADAMTS-4 was determined by qPCR and compared between groups using one-way ANOVA. *p < 0.05, **p < 0.01, and ***p < 0.001

The altered expression of glycosyltransferases contributes to abnormal glycopatterns. Hence, we primarily investigated the alterations in FUTs (fucosyltransferases) in chondrocytes stimulated with TNF-α. As a result, the mRNA levels of FUT3, FUT9, and FUT10 were upregulated in TNF-α-treated chondrocytes compared with control chondrocytes (Fig. 4a). Similar to our findings, FUT10 was upregulated in medial and lateral tibia from OA patients compared with controls (GEO GSE51588) [31] (Fig. 4b). Moreover, the qPCR and immunoblotting results indicated that FUT10 expression is characteristic of OA cartilage (Fig. 4c–e). After transfection, FUT10 expression was significantly reduced (Fig. 4f). The staining results indicated that the number of SA-β-Gal-positive cells was significantly reduced in chondrocytes pretransfected with siRNA-FUT10 compared with TNF-α-treated cells (Fig. 4g, h). Apoptosis was also suppressed by silencing FUT10 (Fig. 4i). The immunoblotting and qPCR results indicated that TNF-α markedly increased the expression of molecules associated with cartilage degeneration, inflammation, and apoptosis, including MMP-13, MMP-9, a disintegrin and metalloproteinase with thrombospondin motifs type 4 (ADAMTS-4), IL-1β, caspase-3, and caspase-8. However, the expression of these OA-associated proteins suppressed the silencing of FUT10. Moreover, pretransfection with FUT10 siRNA attenuated TNF-α-mediated pIκB-α, p-p65, p-p38, and p-JNK expression, suggesting that the downregulation of FUT10 expression suppresses the activation of the NF-κB and p38-JNK pathways in OA chondrocytes (Fig. 4j–l).

### The fucosylation of TNFR1 affected the binding of TNF-α

**Fig. 5:**
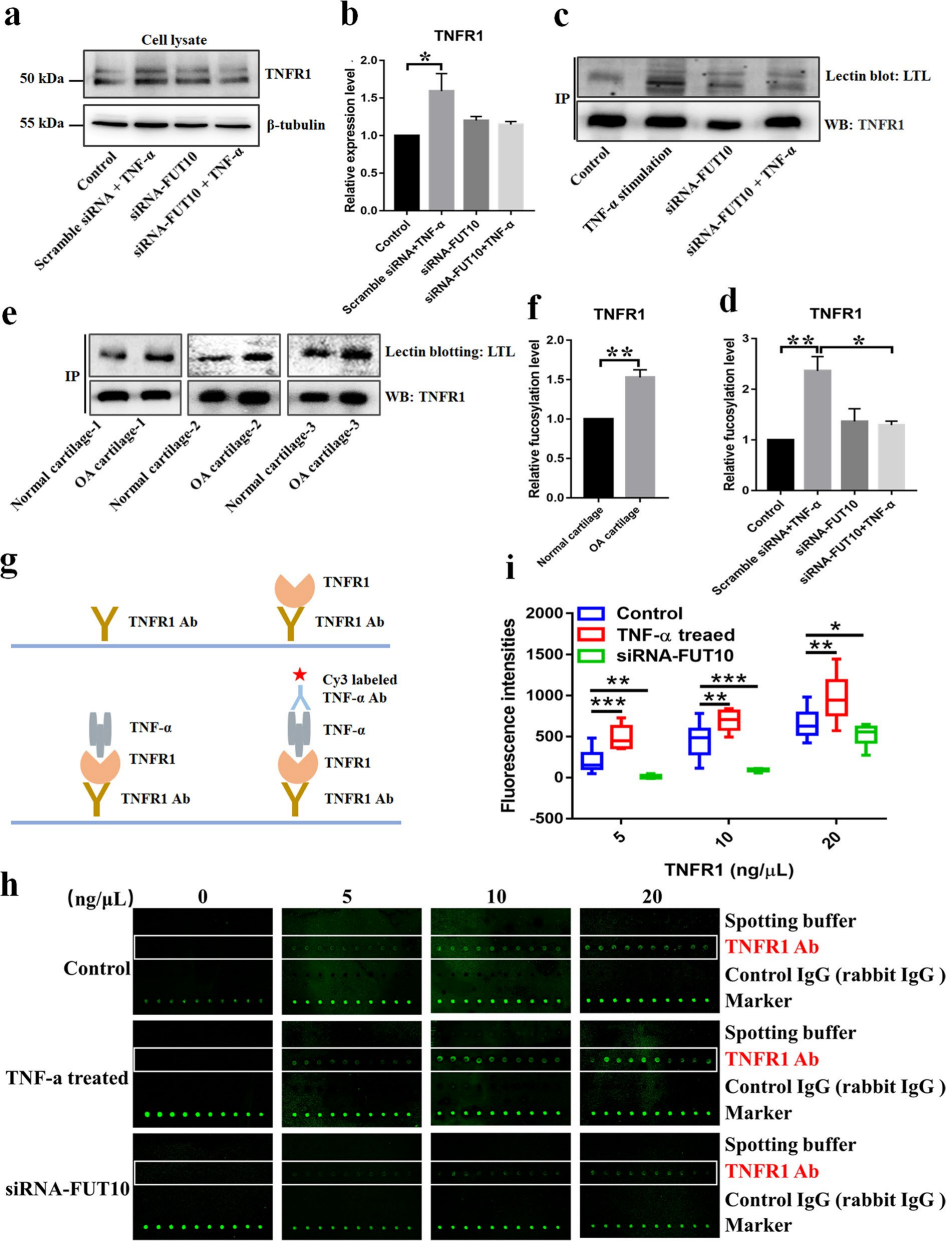
The α-1,3 fucosylation of TNFR1 mediated by FUT10 impacted the binding capacity to TNF-α. **a** Chondrocytes were transfected with siRNA and treated with or without TNF-α for 48 h, and the protein level of TNFR1 was determined by Western blotting. β-Tubulin was used as an internal control. **b** The relative expression level of TNFR1 from three experimental replications was normalized to β-tubulin and compared based on one-way ANOVA. **c** Immunoblot and lectin blot reactivity of TNFR1 immunoprecipitated from chondrocytes treated as described above. **d** The fucosylation of TNFR1 was normalized to the expression of TNFR1 and compared using one-way ANOVA. **e** Immunoblot and lectin blot reactivity of TNFR1 immunoprecipitated from cartilage of OA patients and normal controls. **f** The fucosylation of TNFR1 was normalized to the expression of TNFR1 and compared based on a paired *t test*. **g** A schematic diagram of the fabrication of the TNFR1 antibody microarray. **h** TNFR1 was immunoprecipitated from chondrocytes transfected with siRNA (low level of α-1,3 fucosylation) or induced with TNF-α (high level of α-1,3 fucosylation), and the scanned images were obtained for the analysis of the binding ability of TNFR1 with different levels of fucosylation to TNF-α. The spots of the TNFR1 antibody are marked with white boxes. **i** The fluorescence intensities of spots were extracted by Genepix 7, and the binding ability of TNFR1 was compared based on fold change and *t test*. The data are presented as the mean ± SEM, *p < 0.05, **p < 0.01, and ***p < 0.001

TNFR1 is not only the membrane receptor for TNF-α but also recruits the adaptors FADD and caspase-8 upon binding of the ligand TNF-α to initiate apoptosis [32]. Hence, we explored whether the expression or glycosylation of TNFR1 is altered in TNF-α-stimulated cells. We found that TNFR1 expression was moderately elevated in TNF-α-treated cells compared with control cells but did not find a significant difference in chondrocytes pretransfected with FUT10 siRNA (Fig. 5a, b). The blotting results indicated that α-1,3 fucosylation, but not the levels of the protein itself, was significantly increased in TNFR1 isolated from TNF-α-treated cells compared with controls (Fig. 5c, d). Moreover, the α-1,3 fucosylation of TNFR1 decreased in chondrocytes transfected with siRNA-FUT10, indicating that the downregulation of FUT10 reduces the α-1,3 fucosylation level of TNFR1 without impacting protein expression (Fig. 5c, d). Furthermore, a high level of α-1,3 fucosylation was observed in TNFR1 isolated from OA cartilage compared with normal cartilage (Fig. 5e, f). Taken together, these findings indicated that a high level of α-1,3 fucosylation of TNFR1 was associated with OA. Subsequently, a TNFR1 antibody microarray was used to investigate the role of α-1,3 fucosylation of TNFR1 in the binding ability of TNFR1 to TNF-α (Fig. 5g). This result indicated that the elevated α-1,3 fucosylation of TNFR1 significantly enhanced the binding ability to TNF-α, and downregulation of FUT10 significantly impaired the binding ability (Fig. 5h, i). These findings indicated that the α-1,3 fucosylation of TNFR1 mediated by FUT10 could impact the binding ability of TNFR1 to TNF-α.

## Discussion

OA is the most common type of arthritis worldwide. ECM degradation is a characteristic of OA and correlates with the upregulation of MMPs and elevated levels of proinflammatory cytokines such as TNF-α, IL-1β, and COX-2 [9, 33]. One of the most fascinating aspects of glycosylation is its heterogeneity. The structural heterogeneity of glycans has been observed in various diseases, and it also contributes to the process of OA. In the present study, we observed that several types of glycosylation were altered in OA cartilage from patients and animal models, including elevated levels of α-1,3/6 fucosylation and galactosylation and decreased levels of high mannose-type N-glycans and bisecting GlcNAc. Several studies have indicated that the glycosylation of IgG and synovial fluid is altered during the OA process [34, 35]. In addition, it has been reported that the alteration of glycosylation in a variety of chondrocyte proteins plays an important role in degenerative changes in chondrocytes and contributes to the initiation and progression of OA [36, 37].

Altered fucosylation has been observed in a number of inflammatory conditions, and inhibition of fucosylation could modulate human nucleus pulposus cell protein translation of catabolic enzymes in response to inflammation [38, 39]. Our findings revealed that α-1,3/6 fucosylation is characteristic of OA chondrocytes. Importantly, suppressing the biosynthesis of fucosylation could retard ECM degradation and senescence induced by TNF-α. The Lewis antigen is a fucosylated carbohydrate moiety located at the terminus of N/O-glycans and glycosphingolipids. FUT10 is a member of the α-1,3 fucosyltransferase family, which responds to the synthesis of Lewis X. Sialylated Lewis X is known as an inflammation-associated antigen. We found that FUT10 is overexpressed in OA cartilage and chondrocytes induced by TNF-α; moreover, downregulation of FUT10 could reduce the expression of inflammatory cytokines and ECM degradation. Chronic low-grade inflammation plays an important role in the development of OA. The expression of Lewis X antigen in synovial tissue correlates with histological OARSI grades of OA, which indicates an association of increased inflammatory activity with advanced cartilage degeneration [40]. Sialyl Lewis X promotes synovial lubricin binding to polymorphonuclear granulocytes in an L-selectin-dependent and L-selectin-independent manner and may play a role in polymorphonuclear granulocyte-mediated inflammation in rheumatic arthritis [41].

Proinflammatory cytokines play vital roles in catabolic reactions of arthritic cartilage [42]. As a key inflammatory cytokine, TNF-α is produced by activated synoviocytes, mononuclear cells, or articular cartilage itself. TNF-α promotes the expression of MMPs such as ADAMTS-4, 5, and 7, as well as other inflammatory cytokines, and contributes to cartilage degeneration in OA development [43, 44]. TNFR1 is a cell surface receptor of TNF-α. The binding of TNF to TNFR1 triggers a series of intracellular events that ultimately result in the activation of the NF-κB and p38/JNK MAPK signaling pathways [45]. The N-glycans are pivotal for the TNF–TNFR1 interaction and subsequent NF-κB activation [46]. The α-2,6 sialylation of TNFR1 mediated by ST6Gal-I was found to inhibit TNF-induced TNFR1 internalization and apoptosis [47]. Our results demonstrated that depressed expression of FUT10 not only reduced the α-1,3 fucosylation level of TNFR1 but also impaired the binding capacity to TNF-α and restrained the activation of the NF-κB and p38/JNK MAPK signaling pathways downstream of TNFR1. The activation of the canonical NF-κB pathway is mediated by proinflammatory cytokines (such as TNF-α). As NF-κB heterodimers translocate into the nucleus, the expression of a variety of proteins is triggered, such as metalloproteinases, NF-κB-mediated catabolic cytokines and chemokines, which promote inflammation and apoptosis of OA chondrocytes [48]. The p38/JNK-MAPK pathway was reported to correlate with chronic inflammation. Once activated, downstream transcription factors upregulate the expression of genes relevant to OA, including proinflammatory cytokines and matrix-degrading enzymes such as MMPs [49]. Moreover, the prediction results revealed that Elk-1 and MEF-2 are potential transcription factors for FUT10, which are downstream transcription factors of the P38/JNK-MAPK pathway. Collectively, our results suggested that the inflammatory condition of OA chondrocytes facilitates the expression of TNF-α, which leads to the upregulation of FUT10. Then, the α-1,3 fucosylation of TNFR1 mediated by FUT10 is elevated and promotes the binding ability to TNF-α. Consequently, the NF-κB and P38/JNK-MAPK pathways are activated and promote inflammation and apoptosis of OA chondrocytes. In addition, the transcription factors Elk-1 and MEF-2, which are downstream of the P38/JNK-MAPK pathway, may translocate into the nucleus and upregulate FUT10.

## Conclusions

Our study revealed that the FUT10-mediated increase in α-1,3 fucosylation is characteristic of OA cartilage and chondrocytes. Moreover, the α-1,3 fucosylation level of TNFR1 mediated by FUT10 impacted the binding ability to TNF-α. Silencing the expression of FUT10 impedes activation of the NF-κB and P38/JNK-MAPK pathways and retards ECM degradation, senescence, and apoptosis of chondrocytes induced by TNF-α. Our work indicates a new positive feedback loop of inflammatory conditions/TNF-α/FUT10/α-1,3 fucosylation of TNFR1/NF-κB and P38/JNK-MAPK pathways/proinflammatory processes that contribute to ECM degradation and apoptosis of OA chondrocytes and eventually accelerate the OA process.

## Supplementary Information

**Additional file 1: Table S1**. The information of primers for Real-Time PCR.

**Additional file 2: Table S2.** The sequence information of siRNA against FUT10.

**Additional file 3: Table S3.** The normalized fluorescence intensities from normal and OA cartilage by the lectin microarray analysis based on data of 37 lectins.

**Additional file 4: Table S4.** Altered glycopattern of glycoproteins between sham-operated and OA model cartilage based on data of 14 Lectins giving significant differences.

## Data Availability

The data used to support the findings of this study are available from the corresponding author upon request.
