# Supplementary material for: Elevation of α-1,3 fucosylation promotes the binding ability of TNFR1 to TNF-α and contributes to osteoarthritic cartilage destruction and apoptosis
Source: Arthritis Res Ther. 2022 Apr 29;24:93. doi: 10.1186/s13075-022-02776-z (PMC9052622; doi:10.1186/s13075-022-02776-z)
Supplement: Supplementary file 1 — Additional file 1: Table S1. The information of primers for Real-Time PCR. [file 13075_2022_2776_MOESM1_ESM.docx]

**Table S1. The information of primers for Real-Time PCR**

| Gene name | Symbol Name | Genbank Acc | Primer sequence (5’-3’) | Amplicon size (bp) |
| --- | --- | --- | --- | --- |
| Fucosyltransferase 3 | FUT3 | NM_001097641 | Forward: CTGTCCCGCTGTTCAGAGATG  Reverse: AGGCGTGACTTAGGGTTGGA | 131 |
| Fucosyltransferase 4 | FUT4 | NM_002033 | Forward: CTTCAACTGGACGCTCTCCTA  Reverse: GTTGGTGGTAGTAGCGGACC | 191 |
| Fucosyltransferase 8 | FUT8 | NM_178156 | Forward: AACTGGTTCAGCGGAGAATAAC  Reverse: TGAGATTCCAAGATGAGTGTTCG | 172 |
| Fucosyltransferase 9 | FUT9 | NM_006581 | Forward: CCATTTGGGCAGACCTTTGAC  Reverse: AGAACTGCATGGGATTTGTTGT | 107 |
| Fucosyltransferase 10 | FUT10 | NM_032664 | Forward: GACAGCTACCCCATTATGCTC  Reverse: CGAGGCAGAGGTAAGCTATCTA | 179 |
