# Supplementary material for: Elevation of α-1,3 fucosylation promotes the binding ability of TNFR1 to TNF-α and contributes to osteoarthritic cartilage destruction and apoptosis
Source: Arthritis Res Ther. 2022 Apr 29;24:93. doi: 10.1186/s13075-022-02776-z (PMC9052622; doi:10.1186/s13075-022-02776-z)
Supplement: Supplementary file 2 — Additional file 2: Table S2. The sequence information of siRNA against FUT10. [file 13075_2022_2776_MOESM2_ESM.docx]

**Table S2. The sequence information of siRNA against FUT10.**

| Target gene | Location | Sequence (5′-3′) |
| --- | --- | --- |
| FUT10 | 485 | Antisense: UUGAACAAGGUGAUCACUG |
|  |  | Sense: CAGUGAUCACCUUGUUCAA |
