# Supplementary material for: Elevation of α-1,3 fucosylation promotes the binding ability of TNFR1 to TNF-α and contributes to osteoarthritic cartilage destruction and apoptosis
Source: Arthritis Res Ther. 2022 Apr 29;24:93. doi: 10.1186/s13075-022-02776-z (PMC9052622; doi:10.1186/s13075-022-02776-z)
Supplement: Supplementary file 3 — Additional file 3: Table S3. The normalized fluorescence intensities from normal and OA cartilage by the lectin microarray analysis based on data of 37 lectins. [file 13075_2022_2776_MOESM3_ESM.docx]

**Table S3.** **The normalized fluorescence intensities from normal and OA cartilage by the lectin microarray analysis based on data of 37 lectins^a^**

| **Lectin** | **Specificity** | **Cartilage tissue** | |
| --- | --- | --- | --- |
|  |  | **Normal** | **OA** |
| Jacalin | Galβ1-3GalNAcα-Ser/Thr(T), GalNAcα-Ser/Thr(Tn), GlcNAcβ1-3-GalNAcα-Ser/Thr(Core3), sialyl-T(ST). not bind to Core2, Core6, and sialyl-Tn (STn) | 0.084 ± 0.047 | 0.064 ± 0.036 |
| ECA | Galβ-1,4GlcNAc (type II), Galβ1-3GlcNAc (type I) | 0.033 ± 0.033 | 0.033 ± 0.012 |
| HHL | Manα1-3Man, Manα1-6Man, Man5-GlcNAc2-Asn | 0.044 ± 0.011 | 0.042 ± 0.015 |
| WFA | Terminating in GalNAcα/β1-3/6Gal | 0.028 ± 0.009 | 0.031 ± 0.006 |
| GSL-II | GlcNAc and agalactosylated tri/tetra antennary glycans | 0.014 ± 0.005 | 0.018 ± 0.006 |
| MAL-II | Siaα2-3Galβ1-4Glc(NAc)/Glc, Siaα2-3Gal, Siaα2-3, Siaα2-3GalNAc | 0.014 ± 0.006 | 0.022 ± 0.009 |
| PHA-E | Bisecting GlcNAc, biantennary complex-type N-glycan | 0.017 ± 0.009 | 0.010 ± 0.007 |
| PTL-I | GalNAc, GalNAcα-1,3Gal, GalNAcα-1,3Galβ-1,3/4Glc | 0.015 ± 0.010 | 0.015 ± 0.006 |
| SJA | Terminal in GalNAc and Gal, anti-A and anti-B human blood group | 0.007 ± 0.005 | 0.020 ± 0.012 |
| PNA | Galβ1-3GalNAcα-Ser/Thr(T) | 0.017 ± 0.005 | 0.028 ± 0.008 |
| EEL | Galα1-3(Fucα1-2)Gal (blood group B antigen) | 0.018 ± 0.006 | 0.025 ± 0.010 |
| AAL | α-Fucose | 0.009 ± 0.006 | 0.021 ± 0.011 |
| LTL | Fucα1-3Galβ1-4GlcNAc, Fucα1-anti-H blood group specificity | 0.009 ± 0.005 | 0.019 ± 0.010 |
| MPL | Galβ1-3GalNAc, GalNAc | 0.016 ± 0.003 | 0.027 ±0.008 |
| LEL | LacNAc and poly LacNAc, (GlcNAc)_2-4_ | 0.044 ± 0.017 | 0.040 ± 0.018 |
| GSL-I | αGalNAc, αGal, anti-A and B | 0.013 ± 0.005 | 0.018 ± 0.008 |
| DBA | αGalNAc, Tn antigen, GalNAcα1-3((Fucα1-2))Gal (blood group A antigen) | 0.010 ± 0.004 | 0.018 ± 0.009 |
| LCA | αMan, αGlc | 0.090 ± 0.045 | 0.056 ± 0.043 |
| STL | trimers and tetramers of GlcNAc, core (GlcNAc) of N-glycan | 0.037 ± 0.034 | 0.039 ± 0.031 |
| PTL-II | Gal, blood group H, T-antigen | 0.011 ± 0.007 | 0.015 ± 0.008 |
| DSA | (GlcNAc) 2-4, polyLacNAc and LacNAc (NA3, NA4) | 0.018 ± 0.005 | 0.023 ± 0.010 |
| VVA | terminal GalNAc, GalNAcα-Ser/Thr(Tn), GalNAcα1-3Gal | 0.033 ± 0.009 | 0.040 ± 0.016 |
| MAL-I | Galβ-1,4GlcNAc | 0.007 ± 0.004 | 0.014 ± 0.007 |
| GNA | Manα1-3Man | 0.017 ± 0.007 | 0.026 ± 0.014 |
| NPA | Manα1-6Man | 0.013 ± 0.005 | 0.019 ± 0.005 |
| ACA | Galβ1-3GalNAcα-Ser/Thr (T antigen), sialyl-T(ST) tissue staining patterns are markedly different than those obtained with either PNA or Jacalin | 0.064 ± 0.019 | 0.036 ± 0.011 |
| BPL | Galβ1-3GalNAc, Terminal GalNAc | 0.031 ± 0.016 | 0.045 ± 0.005 |
| PHA-E+L | Bisecting GlcNAc, bi-antennary N-glycans, tri- and tetra-antennary complex-type N-glycan | 0.018 ± 0.005 | 0.018 ± 0.009 |
| SNA | Sia2-6Gal/GalNAc | 0.014 ± 0.005 | 0.017 ± 0.005 |
| RCA120 | β-Gal, Galβ-1,4GlcNAc (type II), Galβ1-3GlcNAc (type I) | 0.064 ± 0.032 | 0.037 ± 0.025 |
| BS-I | α-Gal, α-GalNAc, Galα-1,3Gal, Galα-1,6Glc | 0.015 ±0.006 | 0.022 ± 0.020 |
| PSA | Fucα-1,6GlcNAc, α-D-Man, α-D-Glc | 0.006 ± 0.006 | 0.021 ± 0.011 |
| SBA | α- or β-linked terminal GalNAc, (GalNAc)n, GalNAcα1-3Gal, blood-group A | 0.012 ± 0.004 | 0.022 ± 0.008 |
| WGA | (GlcNAc)_n_ and multivalent Sia | 0.007 ± 0.003 | 0.016 ± 0.007 |
| UEA-I | Fucα1-2Galβ1-4Glc(NAc) | 0.015 ± 0.010 | 0.014 ±0.008 |
| PWM | (GlcNAc)_n_ | 0.014 ± 0.005 | 0.020 ± 0.006 |
| ConA | High-Mannose type N-glycans | 0.120 ± 0.004 | 0.049 ± 0.035 |

^a^ The normalized fluorescence intensities were represented as Mean ± standard error of mean (SEM).
