# Supplementary material for: Elevation of α-1,3 fucosylation promotes the binding ability of TNFR1 to TNF-α and contributes to osteoarthritic cartilage destruction and apoptosis
Source: Arthritis Res Ther. 2022 Apr 29;24:93. doi: 10.1186/s13075-022-02776-z (PMC9052622; doi:10.1186/s13075-022-02776-z)
Supplement: Supplementary file 4 — Additional file 4: Table S4. Altered glycopattern of glycoproteins between sham-operated and OA model cartilage based on data of 14 Lectins giving significant differences. [file 13075_2022_2776_MOESM4_ESM.docx]

**Table S4.** **Altered glycopattern of glycoproteins between sham-operated and OA model** **cartilage based on data of 14 Lectins giving signiﬁcant diﬀerences**

| Lectin Speciﬁcity | | OA model cartilage /sham-operated cartilage^a^ | | | |  |
| --- | --- | --- | --- | --- | --- | --- |
|  |  | 0 week/controls | 4 weeks/controls | 8 weeks/controls | 12 weeks/controls |  |
| PSA | Fucα-1,6GlcNAc, α-D-Man, α-D-Glc | 2.586* | 3.272** | 2.489** | 5.104*** |  |
| AAL | α-Fucose | / | 2.261* | 2.064* | 1.976* |  |
| WGA | (GlcNAc)_n_ and multivalent Sia | / | 1.791* | 2.693* | 2.155** |  |
| STL | Trimers and tetramers of GlcNAc, core (GlcNAc) of N-glycan | 2.284* | 4.325*** | 1.70*** | 2.799** |  |
| DSA | (GlcNAc)_2-4_, polyLacNAc and LacNAc (NA3, NA4) | / | 8.447*** | 2.548*** | / |  |
| UEA-I | Fucα1-2Galβ1-4Glc(NAc) | / | 12.430** | 0.580** | / |  |
| WFA | Terminating in GalNAcα/β1-3/6Gal | 0.589*** | 2.063** | 1.802** | / |  |
| BS-I | α-Gal, α-GalNAc, Galα-1,3Gal, Galα-1,6Glc | / | 3.533** | / | 0.496** |  |
| PTL-II | Gal, blood group H, T-antigen | 1.776** | 1.993*** | / | 0.503** |  |
| LTL | Fucα1-3Galβ1-4GlcNAc, Fucα1-anti-H blood group specificity | / | / | 2.339** | 3.084** |  |
| RCA120 | β-Gal | / | / | 1.635** | 0.535* |  |
| SJA | Terminal in GalNAc and Gal, anti-A and anti-B human blood group |  | 3.296*** | / | / |  |
| PHA-E+L | Bisecting GlcNAc, bi-antennary N-glycans, tri- and tetra-antennary complex-type N-glycan | / | 7.269** | / | / |  |
| SBA | α- or β-linked terminal GalNAc, (GalNAc)n, GalNAcα1-3Gal, blood-group A | / | / | 2.908*** | / |  |
| LCA | αMan, αGlc | / | / | / | 1.990** |  |
| PTL-I | Gal, blood group H, T-antigen | / | 2.820*** | / | / |  |
| BPL | Galβ1-3GalNAc, Terminal GalNAc | / | 1.805** | / | / |  |
| PWM | (GlcNAc)n | / | / | 1.790** | / |  |
| GSL-II | GlcNAc and agalactosylated tri/tetra antennary glycans | / | 1.709* | / | / |  |
| GSL-I | αGalNAc, αGal, anti-A and B | / | / | / | 1.781* |  |
| DBA | αGalNAc, Tn antigen, GalNAcα1-3((Fucα1-2))Gal (blood group A antigen) | 2.064* |  |  |  |  |
| ConA | High-Mannose type N-glycans | / | 0.484* | 0.408* | 0.556* |  |
| PHA-E | Bisecting GlcNAc, biantennary complex-type N-glycan | / | 0.470* | / | 0.619* |  |
| MPL | Galβ1-3GalNAc, GalNAc | 0*** | / | 0.546*** | 0.327** |  |
| LEL | LacNAc and poly LacNAc, (GlcNAc)_2-4_ | 1.867* | / | 0.627** | 0.547*** |  |
| NPA | Manα1-6Man | / | 0.5389** | / | / |  |
| ECA | Galβ-1,4GlcNAc (type II), Galβ1-3GlcNAc (type I) | 0.607** | 0.474* | / | / |  |
| SNA | Sia2-6Gal/GalNAc | 1.997* | 0.441** | / | / |  |
| Jacalin | Galβ1-3GalNAcα-Ser/Thr(T), GalNAcα-Ser/Thr(Tn), GlcNAcβ1-3-GalNAcα-Ser/Thr(Core3), sialyl-T(ST). not bind to Core2, Core6, and sialyl-Tn (STn) | / | 0.403*** | / | / |  |
| MAL-I | Galβ-1,4GlcNAc | / | 0.258*** | / | / |  |
| ACA | T antigen, sialyl-T(ST) tissue staining patterns are markedly different than those obtained with either PNA or Jacalin | / | / | 0.421* | / |  |
| Jacalin | Galβ1-3GalNAcα-Ser/Thr(T), GalNAcα-Ser/Thr(Tn), GlcNAcβ1-3-GalNAcα-Ser/Thr(Core3), sialyl-T(ST). not bind to Core2, Core6, and sialyl-Tn (STn) | 0.435*** | / | 0.492** | / |  |
| EEL | Galα1-3(Fucα1-2)Gal (blood group B antigen) | / | / | 0.385*** | / |  |
| DBA | αGalNAc, Tn antigen, GalNAcα1-3((Fucα1-2))Gal (blood group A antigen) | / | / | / | 0.553*** |  |

^a^ The NFIs of each lectin from OA cartilage was compared with normal controls based on their fold change.

/ did not showed significant difference, * *p* < 0.05, ** *p* < 0.01, and *** *p* < 0.001
